# Constipation in Females

**Published:** 1882-12

**Authors:** 


### CONSTIPATION IN FEMALES.

In a large majority of females troubled with constipation, the constriction of the sphincter ani muscles is the cause. Such constriction is caused by their failing to respond to the demands of nature, and by the delay when the act of defecation, if not impossible, is difficult and painful. Relief can be obtained by using suppository of belladonna at bedtime.

℞ ext. belladonnæ, gr. ss.

Ft. Suppository used before breakfast, ℞. calcined magnesia, ℥ ss., in a lemonade. The belladonna will relax the muscles without causing any constitutional effect—*Martin's Chemists' and Druggists' Bulletin.*

